# Elderly Patients with Hepatocellular Carcinoma Benefit from Liver Transplantation as Much as Younger Ones

**DOI:** 10.1159/000528830

**Published:** 2022-12-26

**Authors:** Jens Mittler, Stefan Heinrich, Martina Koch, Maria Hoppe-Lotichius, Ali Hadian, Arndt Weinmann, Roman Kloeckner, Peter Robert Galle, Hauke Lang

**Affiliations:** ^a^Department of General, Visceral, and Transplantation Surgery, Johannes Gutenberg University Medical Center Mainz, Mainz, Germany; ^b^First Department of Internal Medicine, Johannes Gutenberg University Medical Center Mainz, Mainz, Germany; ^c^Department of Diagnostic and Interventional Radiology, Johannes Gutenberg University Medical Center Mainz, Mainz, Germany; ^d^Institute of Interventional Radiology, University Medical Center Lübeck, Lübeck, Germany

**Keywords:** Liver transplantation, Hepatocellular carcinoma, HCC, Elderly, Septuagenarian

## Abstract

**Introduction:**

The literature on liver transplantation (LT) for cirrhosis-associated hepatocellular carcinoma (cirr-HCC) in elderly patients (≥65 years of age) is scarce. The aim of this study was therefore to analyze the outcome after LT for cirr-HCC in elderly patients in our single-center experience.

**Methods:**

All consecutive patients who underwent LT for cirr-HCC at our center were identified from our prospectively collected LT database and stratified into an elderly (≥65 years) and a younger (<65 years) cohort. Perioperative mortality as well as Kaplan-Meier estimations of overall (OS) and recurrence-free survival (RFS) were compared between age strata. A subgroup analysis was performed for patients with HCC only inside Milan criteria. For further oncological comparison, outcome in the subgroup of elderly LT recipients with HCC inside Milan was also compared to a group of elderly patients undergoing liver resection for cirr-HCC inside Milan extracted from our institutional liver resection database.

**Results:**

Out of 369 consecutive patients with cirr-HCC who underwent LT between 1998 and 2022 at our center, we identified 97 elderly (with a subgroup of 14 septuagenarians) and 272 younger LT patients. 5- and 10-year OS in elderly compared to younger LT patients was 63% and 52% versus 63% and 46% (*p* = 0.67), respectively, while 5- and 10-year RFS was 58% and 49% versus 58% and 44% (*p* = 0.69). 5-/10-year OS and RFS in 50 elderly LT recipients with HCC inside Milan were 68%/55% and 62%/54%, respectively, which compared to 46%/38% (*p* = 0.07) and 26%/14% (*p* < 0.0001) in elderly patients after liver resection for cirr-HCC inside Milan.

**Conclusion:**

Our results in almost 100 elderly patients after LT for cirr-HCC show that older age per se should not be considered a contraindication to LT and that selected elderly patients older than 65 and even 70 years benefit from LT as much as younger ones.

## Introduction

Hepatocellular carcinoma (HCC) is the most frequent primary liver cancer [1]. The incidence of HCC is rising and it is the second highest cause of cancer-related death worldwide. For patients with early-stage HCC arising in cirrhosis (cirr-HCC), liver transplantation (LT) has become the standard treatment as it offers the highest chance of cure. Compared to local ablation and liver resection (LR), LT offers the advantage of not only removing the tumor most radically but also treating the underlying pre-cancerous liver disease from which arises the high risk of recurrence which is estimated to be 60–80% at 5 years [2, 3]. However, LT availability is limited due to a shortage of donor organs. Most countries have implemented a prioritization system and disease-specific eligibility criteria for recipient selection in order to use the scarce resource best and to maximize survival benefit after LT. In Germany as in many other European countries, patients suffering from HCC in cirrhosis are prioritized to LT according to the Milan criteria [4].

With a steady rise in life expectancy in many nations, the population of elderly patients seeking medical help is continuously growing. With regard to HCC, incidence rates of HCC and age are directly correlated until ∼75 years of age in most populations [5]. Although most national LT regulations do not have an explicit age limitation to LT, and LT has been reported even in octuagenarian patients [6], the transplant community seems to be hesitant to serve elderly HCC patients with a liver transplant [7]. The existing literature on these patients is scarce [8]. The aim of this study was therefore to analyze the outcome after LT in elderly (>65 years [subgroup >70 years]) patients with cirr-HCC in our single-center experience.

## Patients and Methods

Our prospectively collected liver transplant center database was searched for all consecutive patients suffering from HCC in cirrhosis who underwent LT between April 1998 and April 2022 at our center. Patients with incidental HCC upon pathologic examination of the explanted liver were excluded from the analysis.

### Age-Groups and Outcome Analysis

Elderly patients were defined as 65 years or older. Within the elderly cohort, a subgroup analysis was performed for patients who were 70 years or older. Overall survival (OS) and recurrence-free survival (RFS) were analyzed as Kaplan-Meier estimations. Survival estimations were censored after 10 years.

To further assess the oncological survival of the elderly transplant cohort with cirr-HCC inside Milan criteria, a comparison was drawn to a control group of elderly patients with cirr-HCC inside Milan criteria who underwent LR at our center. The data of the resection cohort were retrieved from our prospectively collected institutional LR database.

A log-rank test was used to identify statistical significance. The data were significant if *p* was <0.05. Statistical analysis was performed using SPSS software (IBM SPSS Statistics for Windows, version 24.0; IBM Corp., Armonk, NY, USA).

### Evaluation, Listing, Bridging, and LT

Prior to transplant and prior to listing, all patients underwent detailed evaluation examinations to check for medical/surgical suitability for transplantation and eligibility for listing at EUROTRANSPLANT. The evaluation process of medical/surgical transplantability was basically left unchanged over the 24-year study period and only adjusted to the current medical knowledge. Neither our evaluation protocol nor our recipient selection criteria differed between elderly and younger patients. Our recipient selection protocol for all HCC patients included a performance analysis according to the WHO-ECOG performance status scale. An ECOG grade 3 or higher was considered a contraindication to LT. Decision-making was always in accordance with the German LT regulations. In adult recipients, recipient age never was and also currently is not a relevant factor in the German/EUROTRANSPLANT Liver Allocation System. With regard to oncological transplantability and selection criteria in HCC patients, radiologic evidence of extrahepatic tumor spread and/or radiologic evidence of macrovascular portal-venous or hepatic-venous invasion were considered absolute contraindications to listing and transplantation. Since the implementation of a Model for Endstage Liver Disease (MELD)-Score-based liver allocation in Germany in December 2006 with so-called “standard exceptions” for certain diseases, patients with HCC inside the Milan criteria (except for those with very-early-stage HCC, i.e., solitary lesions <20 mm) are prioritized for transplantation by attributing a “standard exception HCC” with an initial exception MELD Score of 22 points (15% mortality) followed by a quarterly increment in exception MELD Score corresponding to a 10% increase in 3 months mortality. Patients with an HCC outside Milan did not qualify for “standard exception” points. They ranked on the EUROTRANSPLANT waitlist according to their laboratory MELD Score and were served with organs from so-called “extended” or “rescue” allocations. Recipient age is not a relevant factor in the EURO HCC bridging while on the waitlist was performed using transarterial chemoembolization, local ablation, or LR as considered appropriate by an interdisciplinary consensus along the 24-year observation period. All decisions regarding the indication to transplant, listing, request for “standard exception,” and oncological bridging were made by the institutional interdisciplinary liver transplant conference. All liver transplant procedures as well as all LR procedures were performed by an experienced surgical team. Recipient age did not have influence on the seniority of the team.

## Results

Between April 1998 and April 2022, a total of 369 consecutive patients suffering from HCC in cirrhosis underwent deceased-donor LT at our center. At the time of LT, 97 patients were 65 years old or older (elderly group), 272 were younger than 65 years. The distribution of the elderly recipients over the 24-year study period did not significantly differ, neither for patients between 65 and 70 years of age nor for septuagenarian recipients (data not shown). The first septuagenarian patient was transplanted in 2005. The patient characteristics are shown in Table 1.

Besides age, elderly patients differed significantly from younger ones only with regard to the cause of underlying liver disease. While the leading etiology of cirrhosis among younger HCC patients was viral hepatitis (55%) and the second most common alcoholic liver disease (31%), this proportion was reversed among elderly HCC patients (alcoholic liver disease (47%) and viral hepatitis (31%)) (*p* < 0.001).

**Fig. 1:**
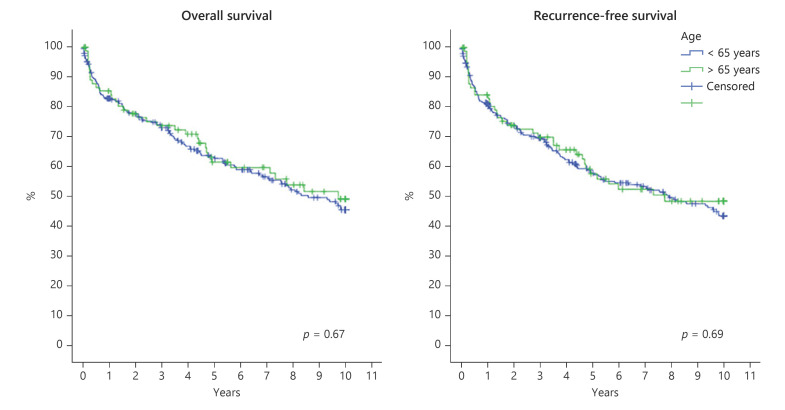
OS (left) and RFS (right) after liver transplantation (LT) of 369 HCC patients, elderly (*n* = 97, green line) versus younger patients (*n* = 272, blue line).

**Fig. 2:**
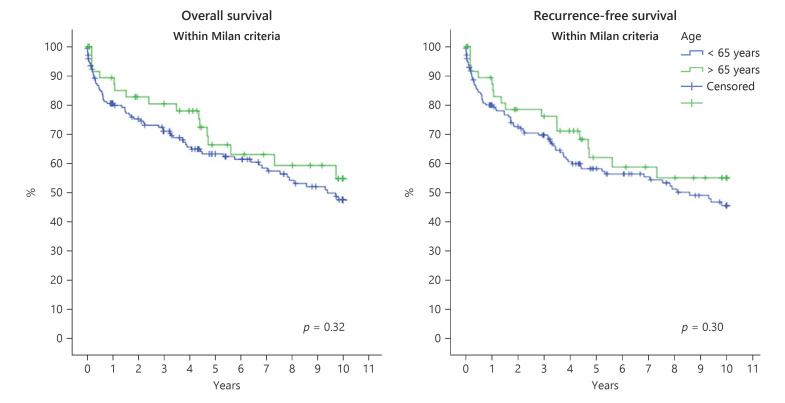
OS (left) and RFS (right) after liver transplantation (LT) of patients with HCC inside the Milan criteria, elderly (*n* = 57, green line) versus younger patients (*n* = 185, blue line).

The 30- and 90-day mortality after LT was 5.1% and 7.2% in the elderly cohort and 4.0% and 7.7% among younger patients, respectively. The overall and RFS of all elderly versus all younger HCC patients regardless of the Milan criteria status are shown in Figure 1. For elderly versus younger patients with HCC only inside the Milan criteria, the overall and RFS estimations are shown in Figure 2.

### Subgroup of Septuagenarian Patients

Within the elderly group of 97 patients, 14 patients were older than 70 years but none older than 75 years. With regard to the severity of the underlying cirrhosis, of note, this septuagenarian subgroup had a significantly lower laboratory MELD Score at time of LT compared to the 75 elderly patients younger than 70 (9 vs. 12, *p* = 0.02). Only one septuagenarian patient was classified to have Child-Pugh grade C cirrhosis. There was one case of perioperative death due to septic multiorgan failure in the septuagenarian subgroup resulting in a 30- and 90-day mortality rate of 7.1% and 7.1%, respectively. The 5- and 10-year OS in this subgroup was 64% and 48%. Ten septuagenarian patients had HCC inside the Milan criteria, their 5- and 10-year OS was 80% and 80%, respectively, and their 5- and 10-year RFS was 60% and 60%.

### Group of Elderly HCC Patients Undergoing LR

The search of our institutional LR database for all consecutive patients between 65 and 75 years of age who underwent LR for cirrhosis HCC at our center identified a total of 77 patients. Out of these 77 patients, 40 (52%) had HCC inside the Milan criteria. The characteristics of these 40 patients are shown in Table 2 together with the characteristics of 57 elderly LT patients with HCC inside Milan. The extent of LR consisted of atypical resections, segmentectomies, and major LRs (>3 segments) in 48%, 44%, and 8%, respectively.

**Fig. 3:**
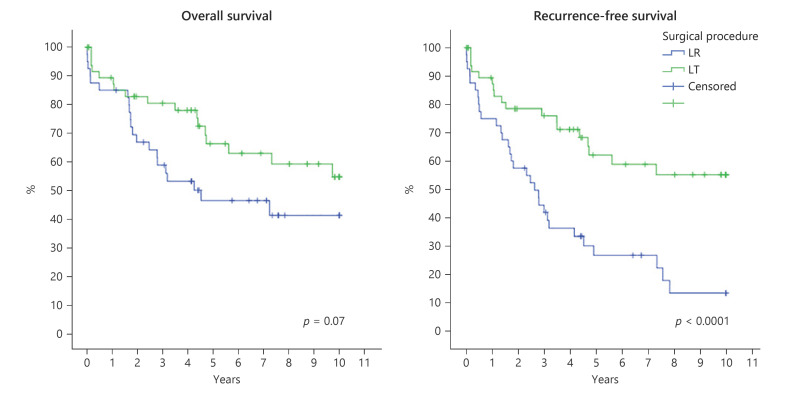
OS (left) and RFS (right) in elderly patients with cirrhosis HCC inside the Milan criteria after liver transplantation (LT) (*n* = 57, green line) versus liver resection (LR) (*n* = 40, blue line).

Compared to the LT cohort, LR patients had significantly less severe cirrhosis as per MELD and CPS Score, had better physical status as of ASA classification, and had larger but less often multifocal tumors with a higher rate of microvascular invasion. LR patients almost never had bridging treatments. The OS and RFS estimations of elderly LT and LR patients with HCC inside the Milan criteria are shown in Figure 3.

## Discussion

While LT is the best oncological treatment for early HCC in cirrhosis, it is also considered the most invasive and drastic treatment modality among its curative alternatives. In elderly patients, the oncological benefit of LT needs to be weighed against an assumed higher age- and comorbidity-associated risk during both the perioperative and postoperative period. Furthermore, the pro-LT argument of recurrence prevention may weigh less in an elderly patient group which starts out with a lesser life expectancy and thus a lower likelihood to experience recurrence. In combination with the omnipresent organ shortage, it may seem ethically controversial to allocate donor livers to elderly patients when younger patients seem to have the greater transplant benefit.

However, overall life expectancy is increasing in many nations due to medical and socioeconomic advancements, and LT has been successfully performed in elderly patients [9]. In our hemisphere, most HCC develop in cirrhosis, the incidence of HCC is increasing [10], and, by 2030, more than 50% of patients with HCC are supposed to be 65 years or older [8, 11]. As a consequence, a growing elderly yet better-aging population is prompting us to re-evaluate the indications of HCC treatments and to re-adjust a maybe outdated perception of the benefit-risk analysis of LT in this patient group.

Our analysis shows that patients with cirrhosis HCC who are 65 years of age or older benefit as much from LT as younger ones. The 5- and 10-year overall and RFS data did not differ between older and younger patients. This was the case when comparing older and younger patients with all HCC (in- and outside the Milan criteria) as well as for those with HCC only inside the Milan criteria. Even in the subgroup of 14 septuagenarian patients, the 5- and 10-year OS was 64% and 48%, respectively. 10 out of these 14 patients had HCC inside the Milan criteria, and their 5- and 10-year OS was 80% and 80%, respectively, and their 5- and 10-year RFS was 60% and 60%, respectively.

The outcome in the elderly transplant cohort in this study is not only comparable to the one in the younger LT cohort, but it also compares favorably to an equally old cohort of LR patients retrieved from our institutional LR database. Despite a significantly better physical performance status, a lesser severity of cirrhosis, and a mostly limited extent of resection (92% atypical or minor resections), LR was associated with a postoperative mortality rate not lower than after LT. In the light of a 30- and 90-day mortality rate of 1.8 and 7.0% after LT, LT was not to be thought of as the high-risk procedure it (maybe) once used to be. Looking at the oncological outcome, the comparison between LT and LR for cirr-HCC inside the Milan criteria showed that LR patients had significantly larger tumors (3.0 vs. 2.4 cm, *p* = 0.006) with a higher rate of microvascular invasion (20 vs. 7%, *p* = 0.045) which were, on the other hand, less often multifocal (5 vs. 40%, *p* = 0.002). The 5- and 10-year OS rate in the elderly LT cohort compared to LR patients was 68% versus 47% and 58% versus 44% (*p* = 0.07) while the 5- and 10-year RFS was 63% versus 28% and 58% versus 15% (*p* = 0.0001), respectively. This survival benefit offered by LT was even more pronounced when considering the median waiting time of 224 days (17–292) between listing and LT in the LT cohort. In contrast, LR patients proceeded routinely to resection without longer waiting times after the decision was made.

As to the limitations of this study, this is a retrospective single-center analysis. In retrospect, it is very difficult to sort out which potential LT patient dropped out for what reason during evaluation or did not stand the “test of time” during bridging while on the waiting list. This flaw can only be overcome by intention-to-treat analyses or even prospective randomized trials which are very difficult to realize in this setting. Especially, the comparison with resection patients − although of the same age and only looking at tumors inside the Milan criteria − is hampered by the fact that LT cohorts always consist of especially selected patients of a more favorable profile with regard to overall condition, comorbidities, and oncological prognosis than that of nontransplant tumor patients. In order to at least partially overcome these limitations for future analysis, our center has adopted an intention-to-treat approach to better assess the risks and benefits of our policy. With the encouraging results in our elderly HCC patients in mind, we will carefully pursue our path of considerately offering a transplant perspective to an aging HCC population. A continuing effort, ideally in conjunction with other transplant centers, is needed to further evaluate this approach.

## Conclusion

Our results in almost 100 elderly patients who underwent LT for cirrhosis HCC at our institution clearly show that older age should not be considered a contraindication to LT in HCC patients per se and that well-selected elderly patients older than 65 and even 70 years can benefit from LT as much as younger ones.

## Statement of Ethics

The study protocol conforms to the Declaration of Helsinki and Good Clinical Practice Guidelines. Ethical approval is not required for this retrospective study in accordance with local or national guidelines. The need for informed consent was waived by Independent Ethics Committee of the State Medical Association.

## Conflict of Interest Statement

None of the authors has any conflict of interest to disclose with regard to this study. Arndt Weinmann received compensations as a member of scientific advisory boards for BMS, Wako, and Sanofi and received travel support from Merck and Servier. Roman Kloeckner consults and advises for Boston Scientific, Bristol-Myers Squibb, Guerbet, Roche, and SIRTEX and is on the speakers' bureau for BTG, Eisai, Guerbet, Ipsen, MSD Sharp & Dohme, and SIRTEX. PRG reports receiving consulting and lectures fees from Adaptimmune, AstraZeneca, Bayer, BMS, Eisai, Ipsen, Lilly, MSD, Roche, and SIRTEX.

## Funding Sources

No funding was received for this study.

## Author Contributions

Jens Mittler, Hauke Lang, and Peter Robert Galle designed the original concept of the study, discussed and modified the study, interpreted data, prepared the first draft of the manuscript, and revised the manuscript drafts. Stefan Heinrich and Martina Koch interpreted data, modified the study, and revised the manuscript draft. Maria Hoppe-Lotichius, Ali Hadian, Arndt Weinmann, and Roman Kloeckner collected and/or assembled data, interpreted data, and performed critical reviews of the manuscript drafts. All authors provided final approval of the manuscript submitted for consideration of publication.

## Data Availability Statement

All data generated or analyzed during this study are included in this article. Further inquiries can be directed to the corresponding author.

## Figures and Tables

**Table 1:** Characteristics of 369 patients undergoing LT for cirrhosis HCC, elderly versus younger patients

	Patients <65 years	Patients ≥65 years	*p* value
n, %	272 (74)	97 (26)	
Age (median, range), years	56.3 (33.6–64.9)	68.9 (65.0–74.2)	**<0.0001**
Male sex (*n*, %)	209 (77)	78 (80)	0.551
Lab-MELD Score at time of LT (median, range)	12 (6–40)	11 (6–40)	0.087
Child-Pugh Score A, B, C (*n*, %)	117 (41), 64 (24), 91 (35)	47 (50), 25 (25), 25 (25)	0.336
Underlying disease (viral/alcoholic/other) (*n*, %)	150 (55), 84 (31), 38 (14)	30 (31), 46 (47), 21 (22)	**<0.001**
HCC inside Milan criteria, outside Milan criteria (*n*, %)	185 (68), 87 (31)	57 (59), 40 (41)	0.121
Largest tumor node, on cross-sectional imagery (median, range), cm	2.5 (1.1–11.0)	2.4 (1.0–10.0)	0.367
Number of tumor nodes (1, 2–3, multiple nodes) (*n*, %)	136 (50), 71 (26), 65 (24)	35 (36), 32 (33), 30 (30)	0.312
Grading Gx/G1/G2/G3/no histology (*n*, %)	16 (6), 46 (17), 156 (57), 43 (16), 11 (4)	5 (5), 18 (19), 57 (58), 15 (15), 2 (2)	0.905
Vascular invasion (yes/no) (*n*, %)	43 (16)	8 (9)	0.154
Pretreatment (*n*, %)	220 (81)	86 (88)	0.137
TACE only (*n*, %)	166 (61)	59 (60)	0.383
Resection only (*n*, %)	6 (2)	1 (1)	
SIRT, RFA (*n*, %)	11 (4)	4 (4)	
Ethanol injection (*n*, %)	3 (1)	2 (2)	
Multiple therapies (*n*, %)	34 (12)	20 (21)	
No evidence of vital tumor in the explant liver (*n*, %)	40 (15)	20 (21)	0.238
Waiting time (listing to LT)	191 (1–391)	167 (13–259)	0.672
Follow-up (median, range), days	1,639 (98–7,195)	1,702 (87–6,471)	0.881
Mortality within 30 days (*n*, %)	11 (4.0)	5 (5.1)	0.062
Mortality within 90 days (*n*, %)	21 (7.7)	7 (7.2)	0.59

LT liver transplantation; HCC, hepatocellular carcinoma; Lab-MELD Score, laboratory Model for Endstage Liver Disease Score; TACE, transarterial chemoembolization; SIRT, selective internal radiation therapy; RFA, radio frequency ablation.

**Table 2:** Characteristics of elderly patients with HCC inside Milan criteria, LT versus LR

*n*	LT patients ≥65 years (inside Milan) 57	LR patients ≥65 years (inside Milan) 40	*p* value
Age (median, range), years	67.5 (65.2–74.2)	68.4 (65.0–75.8)	0.12
Male sex (*n*, %)	43 (76)	31 (78)	0.868
Lab-MELD Score (median, range)	11 (6–40)	7 (6–10)	**<0.001**
Child-Pugh Score A, B, C (*n*, %)	27 (48), 16 (28), 14 (24)	40 (100), 0 (0), 0 (0)	**<0.001**
Underlying disease (viral/alcoholic/other) (*n*, %)	16 (28), 27 (48), 12 (24)	17 (42), 14 (35), 9 (22)	0.293
ASA classification 2, 3, 4 (*n*, %)	0 (0), 45 (79), 12 (21)	13 (32), 22 (55), 1 (2.5)	**<0.001**
Largest tumor node on pre-op imagery (median, range), cm	2.4 (1.1–5.0)	3.0 (1.5–5.0)	**0.006**
Number of tumor nodes (1, 2-3) (*n*, %)	34 (60), 23 (40)	38 (95), 2 (5)	0.002
Grading G1/G2/G3/Gx/no histol. (*n*, %)	11 (20), 30 (52), 11 (20), 4 (7), 1 (2)	7 (17), 26 (65), 7 (17)	0.715
Microvascular invasion (yes) (*n*, %)	4 (7)	8 (20)	**0.045**
Pretreatment (*n*, %)	46 (80)	3 (7.5)	**<0.001**
TACE only (*n*, %)	26 (44)	1 (2.5)	
Resection only (*n*, %)	1 (2)	1 (2.5)	
SIRT, RFA (*n*, %)	3 (6)	0 (0)	
Ethanol injection (*n*, %)	2 (4)	0 (0)	
Multiple therapies (*n*, %)	14 (24)	1 (2.5)	
No evidence of vital tumor in the explant/specimen after pretreatment (*n*, %)	11 (19)	0 (0)	
Waiting time	224 (17–292)	−	
Follow-up (median, range), days	1,622 (7–3,650)	1,153 (1–3,650)	0.199
Mortality within 30 days (*n*, %)	1 (1.8)	3 (7.5)	0.08
Mortality within 90 days (*n*, %)	4 (7.0)	5 (12.5)	0.482

HCC, hepatocellular carcinoma; LT, liver transplantation; LR, liver resection; Lab-MELD Score, laboratory Model for Endstage Liver Disease Score; TACE, transarterial chemoembolization; SIRT, selective internal radiation therapy; RFA, radio frequency ablation.
